# A Mobile Breast Cancer Survivorship Care App: Pilot Study

**DOI:** 10.2196/cancer.8192

**Published:** 2017-09-26

**Authors:** Janet Baseman, Debra Revere, Laura-Mae Baldwin

**Affiliations:** ^1^ Department of Epidemiology University of Washington Seattle, WA United States; ^2^ Department of Health Services University of Washington Seattle, WA United States; ^3^ Department of Family Medicine University of Washington Seattle, WA United States

**Keywords:** breast neoplasms, data collection, feasibility studies, mobile apps, survivors, telemedicine

## Abstract

**Background:**

Cancer survivors living in rural areas experience unique challenges due to additional burdens, such as travel and limited access to specialists. Rural survivors of breast cancer have reported poorer outcomes, poorer mental health and physical functioning, and lower-than-average quality of life compared to urban survivors.

**Objective:**

To explore the feasibility and acceptability of developing a mobile health survivorship care app to facilitate care coordination; support medical, psychosocial, and practical needs; and improve survivors' long-term health outcomes.

**Methods:**

An interactive prototype app, SmartSurvivor, was developed that included recommended survivorship care plan components. The prototype's feasibility and acceptability were tested by a sample of breast cancer survivors (n=6), primary care providers (n=4), and an oncologist (n=1).

**Results:**

Overall, both survivors and providers felt that SmartSurvivor was a potentially valuable tool to support long-term survivorship care plan objectives. Portability, accessibility, and having one place for all contact, treatment, symptom tracking, and medication summaries was highly valued.

**Conclusions:**

Our pilot study indicates that SmartSurvivor is a feasible and acceptable approach to meeting survivorship care objectives and the needs of both breast cancer survivors and their health care providers. Exploration of mobile health options for supporting survivorship care plan needs is a promising area of research.

## Introduction

Cancer patients are surviving longer. Since the early 1990s, the overall cancer death rate has steadily declined and the 5-year survival rate is now 69%, up from 49% in the 1970s [1]. Survivorship care planning aims to meet the need for ongoing, long-term surveillance and management of cancer survivors and to promote wellness and healthy lifestyle behaviors [2-4].

Most breast cancer survivors who do not die of their cancer may die from conditions that can be managed and are modifiable through lifestyle changes (eg, respiratory and heart disease) or screening (eg, colon cancer) [5]. Among breast cancer survivors, a high degree of self-efficacy—the belief that one can control challenging environmental demands by taking adaptive action—is associated with increased self-care behaviors, decreased physical and psychological symptoms, and increased quality of life after treatment [6-8]. Evidence suggests that survivor self-efficacy can be enhanced with appropriate, tailored, self-management support and making lifestyle changes to promote health, well-being, and survival [9].

One strategy to support survivors is through the development of comprehensive survivorship care plans (SCPs), which offer a blueprint for long-term management and a means to support follow-up care coordination and communication. Improvements in the quality of cancer patient-provider communication are associated with more participatory decision making, improved medical information seeking and understanding of information, improved facilitation of information exchange, reduced depression and other negative psychosocial needs associated with survivorship, and improved quality of life [8,10-13]. Increasingly, cancer survivors are viewed as part of the population of patients considered chronically ill and in need of care that is integrated within the wider context of health, prevention, and well-being. Improved provider-provider communication and information sharing can enable cancer-related care as a component of overall prevention and wellness, empower patients with the skills and resources they need for tackling cancer-related problems, and enhance survivor self-efficacy [8].

However, SCPs are not consistently received by cancer patients or their care providers [14-16], as recommended by the Institute of Medicine (IOM) [3], the Commission on Cancer standards for survivorship care planning [17], and services such as Journey Forward's Survivorship Care Plan Builder [18]. In addition, for survivors living in rural areas there are additional and unique challenges and barriers in survivorship care planning, adherence, and coordination that lead to poorer outcomes, including higher psychological morbidity, poorer quality of life, poorer physical functioning, and increased complaints of cancer-specific symptoms [19,20]. Rural survivors also experience barriers regarding access to treatment, medical providers and health information, psychosocial adjustment and coping, and social and psychological support services, in part due to increased travel for medical services with associated burdens of time, cost, and discomfort [21-24]. The prevalence of numerous health-compromising behaviors, such as smoking, health-related unemployment, and physical inactivity, are significantly higher in rural survivors [25]. Rural survivors are also less likely to seek mental health services and cancer support groups [20,26]. In addition, specific to breast cancer survivors, those living in rural areas are more likely to report experiencing distress, high levels of depression and hopelessness/helplessness, and lower-than-average quality of life compared to urban survivors of breast cancer [27,28].

The unique challenges faced by rural survivors require unique and targeted interventions that mitigate survivorship planning and care barriers associated with residing in rural locations [3,25]. With the growth of mobile technologies in rural areas, opportunities have grown for mobile health (mHealth) venues to enhance communication between patients and providers and improve distribution of cancer SCPs [29]. For survivors living in rural settings, mHealth technologies have the potential to facilitate care coordination; support medical, psychosocial, and practical needs; and improve survivors' long-term health outcomes [30,31]. Some research has reported that rural breast cancer survivors are more likely to prefer electronic modes of communication for submitting questions about SCPs to care providers than urban survivors [8]. Research has also reported that remotely accessible mechanisms—phone, Internet, and email—may be highly effective in meeting rural breast cancer survivors' physical and psychosocial needs [11].

The most common format for distributing SCPs is as a written, hard-copy format. Characteristics of static content and lack of portability may contribute to the perception of their uncertain value and limited utility in easing the transition from active treatment into survivorship by both survivors and providers [32]. A systematic review of survivors' experiences using SCPs recommended that SCPs should be “living” documents in electronic formats that are portable and can be modified and readily available to all stakeholders [33]. Supporting the mobile delivery and “anytime” access to the SCP on a mobile phone has the potential to meet this requirement; as the survivor moves along the survivorship continuum and her or his needs change [34], updates and modifications need to be made. A flexible, reprogrammable, portable tool could accommodate these changing circumstances and needs and ultimately offer a unique approach to handling a survivor's evolving status. At the same time, this tool could facilitate time-sensitive communication of information to support collaborative decision making between survivors, their oncologists, and primary care physicians.

This study explores the feasibility and acceptability of an mHealth app, SmartSurvivor, that incorporates recommended components of a survivorship care plan into a mobile survivorship monitoring and management app for rural breast cancer survivors. It also collects system development requirements and feature enhancements for ensuring the app will enhance survivor self-efficacy, improve patient-provider communication, support adherence to SCP recommendations, and promote decision making among rural breast cancer survivors and their providers.

Between September and December 2014, we undertook a pilot study to evaluate whether converting an SCP into a mobile app that includes IOM-recommended content for survivorship care planning is a feasible and acceptable option for breast cancer patients who have completed active treatment. We also evaluated whether this mobile app could be a tool that can assist providers in their care decision making with breast cancer survivors.

## Methods

### Prototype Development and Design

**Figure 1:**
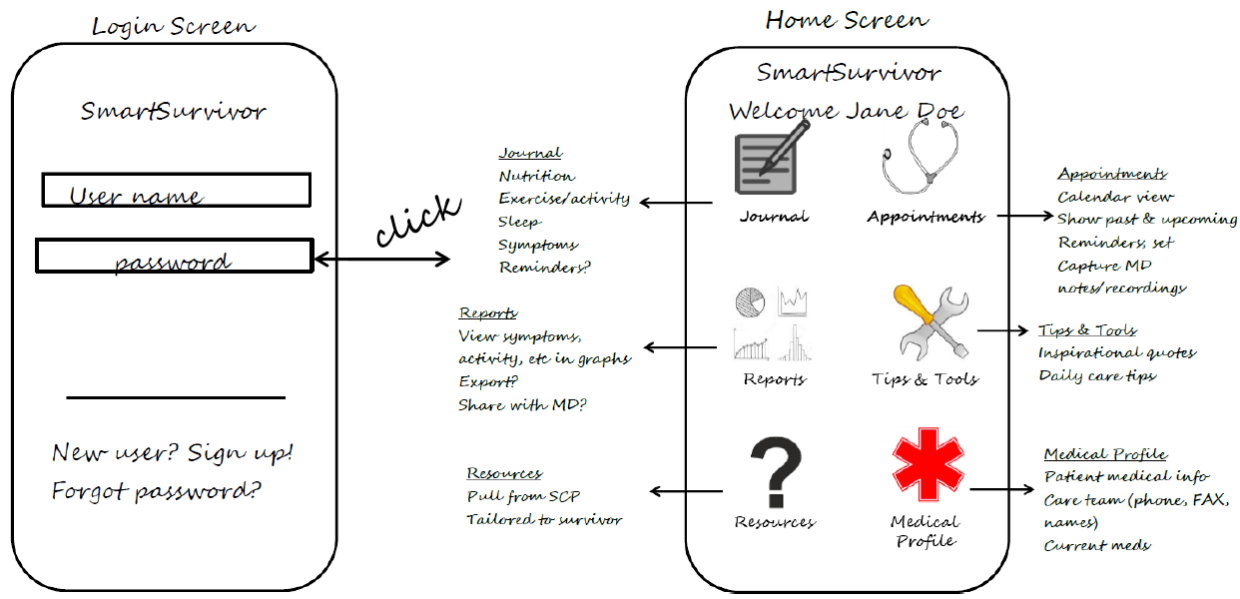
Prototyping development.

Development was undertaken in two phases. In the first early design phase, two members of our research team and a graphic designer used paper prototyping and storyboarding to assess design ideas (see Figure 1) and to block out navigation of IOM-recommended SCP components on individual screens [35]. To situate our design as realistically as possible, in addition to a literature review, we utilized Zapka et al's *Cancer Treatment and Transition to Survivorship* Case Scenario, which was developed to highlight the multilevel issues encountered in cancer survivorship and the challenges they present to designing and testing interventions for this population [36]. Walkthroughs with the paper mock-ups and use-case scenario were conducted before a final paper prototype was approved by the research team.

**Figure 2:**
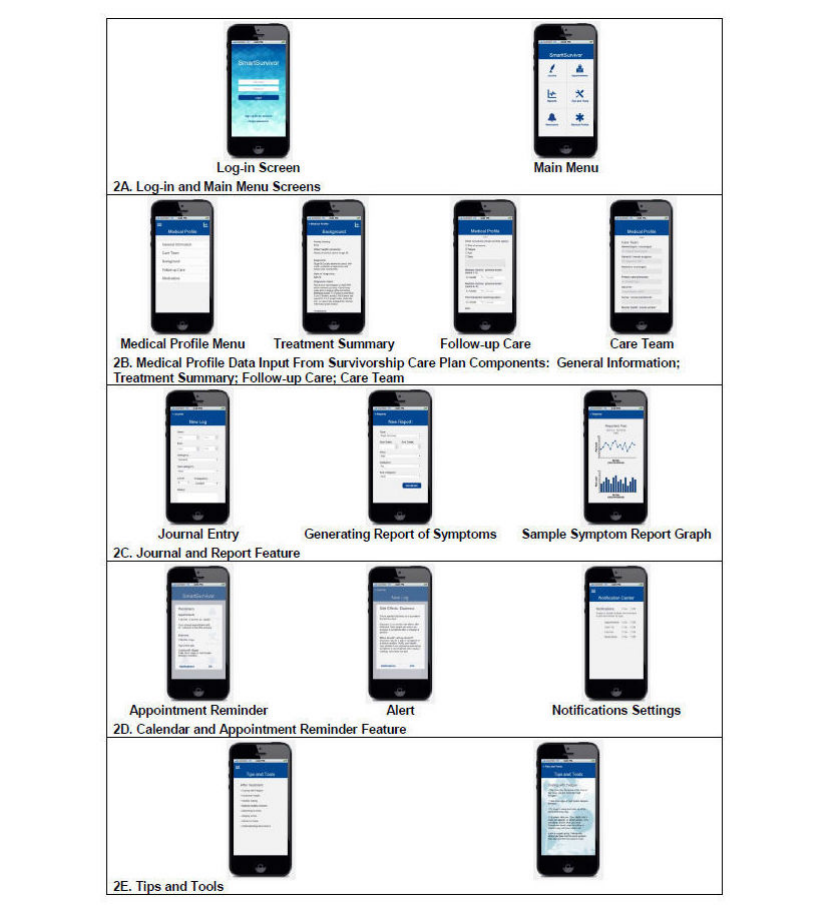
SmartSurvivor prototype screens.

In the second phase, we utilized Axure (Axure Software Solutions), a layer-based wireframe, rapid-prototyping software tool that allows linkages and dynamic interactions between pages and screens presented in a mobile phone app to simulate real-time interactions and navigation. Axure facilitated assessment of features such as flexibility of drop-down lists and movement between screens, as well as readability, navigation, and size of text fields that could impact acceptability of the app [37]. The tool generates a downloadable mobile phone app and HTML website, creating a functional prototype with the look and feel of the actual app without requiring any coding. Based on our paper prototyping, the following were built as interactive screens into Axure (see Figure 2 for screenshots):

1. Log-in Screen and Main Menu (see Figure 2 A).
2. Medical Profile, which includes General Information; Care Team (past, current); Treatment Summary (diagnosis, radiation treatment summaries, etc); and Follow-Up Care (eg, ongoing toxicities to track, wellness/concerns, recommended follow-up schedule/frequency, etc) (see Figure 2 B).
3. Journal and Reports component, which includes a tracking tool for self-monitoring and output of logged Journal data (see Figure 2 C).
4. Calendaring link-in for Reminders, Appointments, and Notifications, including an Alerting function linked to Follow-Up Care and Journal logs to, for example, issue an alert if the survivor is dizzy for 3 days (see Figure 2 D).
5. Tips and Tools that deliver tailored tips to survivors based on data input into their Journal (Figure 2 E).
6. A mobile phone audiotaping link-in for documenting notes, appointment questions, etc.

“Mock” patient health information entered into SmartSurvivor was extracted from samples provided by Journey Forward's Survivorship Care Plan Builder [18]. For testing, the final app prototype was uploaded on a mobile phone and the HTML version was loaded onto a laptop on a website that did not require Internet connectivity.

### Ethics

This study received University of Washington Institutional Review Board approval as an exempt study. Informed consent was obtained from all individual participants included in the study.

### Recruitment

Given the objective of supporting communication and coordination of care, we included both breast cancer survivors and providers in our testing sample to ensure the feasibility of SmartSurvivor for meeting the needs of all end users while minimizing barriers to its utility, feasibility, and acceptability. A convenience sample of breast cancer survivors (n=6), primary care providers (n=4), and an oncologist (n=1) were recruited to participate as testers through contacts of one of the study's investigators. Survivors ranged between 2 months and 5 years postactive treatment and lived in an urban area. All primary care providers had prior experience working in rural settings in which they saw breast cancer patients for ongoing care, including cancer surveillance. All testers owned a mobile phone.

### Testing

Testing sessions lasted between 45 and 60 minutes and were led by a research team member with expertise in usability testing accompanied by a notetaker. Sessions were held at the location and time convenient for each tester and utilized the talk- or think-aloud protocol, which is a widely used method for capturing the usability of an mHealth app [38]. After a brief introduction to the project, testers were walked through the prototype and then allowed to interact with the mobile phone version. Through informal questioning, survivor and provider testers were asked to comment on SmartSurvivor's features (eg, logging and reporting); utility for supporting SCP objectives, survivorship care, and self-management; resources; and overall ease of use. While navigating through the screens, all testers were asked to talk aloud about their expectations regarding interactions with buttons and drop-down menus, navigation, and clarity of information presented in each of the components. Provider testers were also asked to comment on how SmartSurvivor might integrate with their care delivery strategies. At the conclusion of the session, testers received a US $25 gift card for their participation.

Following each session, the testing team collated their notes and observations in a debriefing session. The final set of notes was summarized and the content analysis method [39], a qualitative data analysis strategy used to generate recommendations from moderated discussions [40], was used to evaluate and synthesize the testing sessions into themes.

## Results

### Thematic Outcomes

Six primary themes emerged from the analysis (see Textbox 1) and are detailed below.

Overall, both survivors and providers were positive about the value of using SmartSurvivor to support survivorship care objectives, thought it would be easy to use, and viewed SmartSurvivor as a way to make the SCP more accessible and useful. All testers were familiar with, and comfortable using, a mobile phone and regularly accessed the Internet on their mobile phones to access information, including health information.

### Theme 1: SmartSurvivor Provides One-Stop Shopping

Having one place to file all contact and treatment information is supportive of coordination of care and provider-provider communication. Overall, survivors were enthusiastic about having one location as a repository for medical team contact information, treatment records, insurance numbers, etc. SmartSurvivor was seen as a good *memory aid*, as illustrated in the following quotes:

I have a hard time remembering all the doctors I have, when I've seen them.

I'm always asked what medications I'm on so this would make it a lot easier.

Providers also saw the ability to obtain treatment and medication summaries from one source as useful, stating it would streamline the fact-finding portion of an appointment:

If I had a patient experiencing continued dizziness, being able to see exactly what treatment she's had will help me figure out if this is a side effect of treatment or if it's unrelated.

Providers also saw SmartSurvivor as a way to support coordination of care with specialists when consultation about the patient is needed.

### Theme 2: Survivors and Providers Are Empowered by Better Tracking and Communication

The combination of Journaling symptoms and their output—Reports—may improve self-efficacy for management of survivorship needs and be supportive of improved patient-provider communication and provider decision making. Survivors not only need to track symptoms and remember appointments, they are encouraged to engage in wellness activities, monitor their sleep and eating, and track their mood. Some survivors carried notebooks, some used the *Notes* function in their mobile phones, and one had developed an elaborate system of sticky notes that were entered into a spreadsheet at the end of the day to keep track between medical visits. All survivors stated that their current methods are inadequate to their ongoing surveillance and monitoring needs. SmartSurvivor was seen as a way to improve self-management to support SCP objectives with a tool for tracking symptoms, wellness activities, mood, etc:

I have to be my own patient advocate all the time and this will help me track what I need to track. And then, when I see the doctor I can give her something concrete like this graph, instead of saying, “yeah, I was really tired last week,” I can show her how tired, for how long.

I'm on medication that increases my risk of ovarian cancer so I need to track any spotting. This would make it much easier than the piece of paper I'm using now.

In addition, survivors stated that being able to see results, notice trends, and track patterns would be very useful.

**Key findings from pilot-testing SmartSurvivor with survivors and providers.**

Theme 1: SmartSurvivor provides one-stop shopping.

Theme 2: Survivors and providers are empowered by better tracking and communication.

Theme 3: Portability is a plus.

Theme 4: Interoperability/integration of SmartSurvivor with other information sources is a concern.

Theme 5: Survivors are uncertain about being reminded.

Theme 6: SmartSurvivor needs to be tailored for rural users.

For providers, the Report function was seen as valuable for accurate and informative patient reporting, as well as for saving time during a visit:

Seeing a graph of what symptoms the patient is having and when is more informative.

We have so little time during an appointment; this would help me spend more time with my patients.

It was also seen as informing decision making about a patient's care:

Let's say she's experiencing pain and swelling under her right arm. It's on the graph but I also see she started a new exercise regimen. Instead of sending her back to her surgeon 250 miles away in a snowstorm, we might explore other options first, like get a CT or MRI scan locally. Plus, I could easily coordinate with her surgeon about this option since all the contact information is right here.

Both providers and survivors stated that being able to create overlays of reports, for example, a graph that combines pain with sleep or exercise with mood, would be even more useful than individual reports. The SmartSurvivor email function that allows the survivor to email an output of a Report in Excel or graph form in advance of a medical appointment was seen as supporting communication and coordination of care by both survivors and providers. Both survivors and providers viewed the ability to record questions and comments before or after a medical appointment as improving patient-provider communication; survivors appreciated the ability to document questions and concerns before medical visits and providers thought this feature would encourage more productive communication during visits.

### Theme 3: Portability Is a Plus

Within this sample of mobile phone users, the phone is carried everywhere and used routinely as part of everyday living. In comparison, the standard, paper-based SCP is a heavy notebook that is a burden to carry, as reflected in the following quote:

I was told I shouldn't lift more than 5 pounds after my surgery so this [notebook] just sat on the table for months!

Because their mobile phones are always available, survivors liked the idea of having an app that is not only convenient but very portable for meeting their survivorship care needs. Primary care providers reported their survivor patients rarely brought the entire SCP notebook to an appointment even when first transitioning from active treatment, but occasionally brought a subset of its pages.

### Theme 4: Interoperability/Integration of SmartSurvivor With Other Information Sources Is a Concern

Some survivors are using mHealth tools like MyFitnessPal to track their diet and exercise. Although not developed with survivors in mind, having the capacity to link or integrate data from these different apps with SmartSurvivor would be beneficial and enhance its use as the primary support tool. Survivors who are seen in multiple health care systems also mentioned interoperability as beneficial:

I go to three different doctors and none of them can see the other's records so I have to coordinate my own care.

Providers also brought up interoperability between health care systems as a possible barrier and expressed concerns about data quality if SmartSurvivor information were input by hand versus through the electronic medical record system.

### Theme 5: Survivors Are Uncertain About Being Reminded

In addition to the Journal and Report features, the Reminders/Notifications/Tips functions were seen as important to ongoing surveillance for both survivors and providers. In general, reminders regarding follow-up tests and appointments were viewed as useful, but this appeared to depend on status within the survivorship continuum. Survivors less than 3 years postactive treatment were positive about Reminders. Survivors over this point stated they might turn this feature off because they did not want to be reminded about their cancer. In fact, one survivor close to her 5-year anniversary date stated, “I don’t want to obsess over my care, over my cancer. I just want to live my life.”

Overall, providers also found the Reminder function as useful, but only if recurring reminders could be recalibrated easily if an appointment is missed. This would be particularly important for rural survivors who frequently need to reschedule appointments due to transportation issues (eg, weather disruptions and distance). Providers also noted that background information would be helpful in deciding whether a routine appointment could be completed locally rather than by a specialist who practices hundreds of miles away.

### Theme 6: SmartSurvivor Needs to Be Tailored for Rural Users

Several issues specific to rural survivors' needs were brought up by providers. Primary care providers reported that even though the recommended health literacy level for the SCP is around 8th grade, survivors in rural settings often have a lower literacy level and a large proportion are not native English language speakers. For patient education, providers reported using photos, visual cues, and simplified language. Another issue is tracking symptoms, wellness, mood, etc; again, survivors with lower literacy levels may have different needs. One provider stated the following:

It has to be simple for most of my rural patients. And I need to instruct a patient in how to record symptoms, to record simple events and at the time (or closest to the time) they're actually occurring. If the app had a way to click to capture symptoms, then you could potentially generate time of day and frequency just from that. Simpler is better for tracking; if it gets too hard, my patients won't use it.

## Discussion

### Principal Findings

We successfully pilot-tested a prototype SmartSurvivor app that was both feasible and acceptable to a small sample of urban breast cancer survivors and health care providers and that could serve as the foundation for developing a tool to support rural breast cancer survivors. While some mHealth tools have been developed for survivors [29], these are limited to areas such as exercise- and diet-monitoring apps and lack concordance with comprehensive survivorship care planning.

We identified key features that will be important to include in further development, as well as exploration of the SmartSurvivor app with a larger sample of both survivors and providers, including the following:

1. Simplifying the data input process for patients by (a) improving Journaling to include drop-down menus or other features that streamline the data input process and (b) enabling auto-capture of date and time.
2. Improving the data output feature by creating the ability to build overlays of individual graphs, for example, sleep, exercise, and pain.
3. Establishing interoperability between SmartSurvivor and other tools by (a) creating linkages between SmartSurvivor and electronic health record systems to increase confidence that clinical data (ie, appointments, medication lists and changes, and test results) are accurate and (b) enabling linkages with patient tracking tools such as Fitbit, MyFitnessPal, etc.

Those living in rural areas experience unique challenges in their survivorship care [19-28]. Although mobile health technologies have the potential to mitigate some of those challenges, the unique needs of rural survivors identified in this study, such as health literacy levels, need to be addressed in building an mHealth app for this population.

We believe that this pilot study establishes the foundation for future work on SmartSurvivor that will include a larger sample of rural survivors and providers to explore efficacy. A proposal is currently in process to conduct a randomized trial that will (1) compare SmartSurvivor use versus *usual care* (ie, paper-based SCP alone) on patient-reported self-efficacy; (2) determine the effect of SmartSurvivor use on adherence to SCP recommendations, quality of life, patient-provider communication, and care coordination as compared to usual care; and (3) explore the utility of SmartSurvivor for informing health care providers' decision making around clinical care and care coordination for their breast cancer survivor patients.

### Limitations

A limitation of this work is the absence of rural breast cancer survivors, as well as inclusion of a single oncologist. However, including primary care providers with experience practicing in rural settings helped us capture some additional features to include in SmartSurvivor. This also helped identify a baseline of high-quality functional design requirements that will align the app with the needs of all its end users and minimize barriers to its use.

### Comparison With Prior Work

Our findings explore an area—mobile support for rural breast cancer survivors who have completed active treatment—that has received little attention in research studies and few efforts to address. The literature recommends that SCPs become portable, flexible, and easy to access and update as survivors' needs change along the survivorship continuum [3,17]. The logical evolution of support for survivors is to identify the requirements for, and explore the feasibility and acceptability of, building an mHealth tool to meet this need. However, no work has been conducted in this area for the largest group of cancer survivors.

Feasibility and acceptability of an mHealth tool is only a first step. A factor in the development of any mHealth tool is its content. A recent review of the content available within mobile phone apps targeting cancer support reported that only 48.8% of mHealth tools surveyed had been developed by health care organizations and professionals [32]. While our work did utilize providers and cancer specialists in reviewing SmartSurvivor, we anticipate development of content after this point will also include these user groups, as well as rural breast cancer survivors.

For those in rural settings where barriers to optimal care and lower health outcomes have been well-documented [3,20-26], a mobile SCP is a promising intervention. However, it is unknown how mHealth technologies may be leveraged to support the specific and vulnerable group of breast cancer survivors living in rural settings. The work described in this paper addresses a significant gap in the cancer survivorship field.

### Conclusions

Making the SCP accessible, portable, and *always available* has the potential to empower survivors to actively engage in planning, monitoring, and following health behavior guidelines throughout the survivorship trajectory. SmartSurvivor will provide a unique approach to survivorship care planning as a repository for the survivor's unique history and self-management needs, as well as a mechanism for sharing this information with her care team. This approach is responsive to a survivor's fluid and evolving needs, accommodating these changing circumstances and needs. At the same time, this approach facilitates time-sensitive communication of this information to support collaborative decision making between survivors, their oncologists, and primary care physicians.

## Abbreviations

IOM: Institute of Medicine
mHealth: mobile health
SCP: survivorship care plan
